# Supplementary material for: Contradictions and possibilities for change: Exploring stakeholder perspectives of Canada’s Feminist International Assistance Policy (FIAP) and their connection to a future for global health
Source: PLOS Glob Public Health. 2024 Nov 8;4(11):e0003877. doi: 10.1371/journal.pgph.0003877 (PMC11548757; doi:10.1371/journal.pgph.0003877)
Supplement: S1 Text — (DOCX) [file pgph.0003877.s001.docx]

## S1_Text

## Interview Guide

*Total time required:* 40-70 minutes

*Breaks:* As many as necessary

*Introduction*

Hello, thank you for taking the time to speak to me today. I am a PhD student in the Global Health department at McMaster. I am conducting a study that explores stakeholder perspectives of Canada’s Feminist International Assistance Policy (FIAP) and how the policy shapes adolescent sexual and reproductive health (ASRH) projects. Through today’s interview I am hoping to learn about your perspective on the FIAP, how it shapes ASRH projects and your thoughts on how the Canadian government can better fund and support organizations implementing projects aligned with local community goals.

Would it be ok if I recorded the interview? The recording will be confidential and not shared with anyone, I will be storing notes from our conversation today in a secure place so they cannot be lost or accidentally made public. I will also be removing any identifying information from the data, so the analysis will be completed with your privacy and confidentiality as a priority.

(if consented too …). This interview will consist of a few semi-structured questions, so I may ask follow up questions throughout. You may choose not to answer any of the questions. We can take breaks whenever you’d like and if you’d like to end the interview at any point, that is okay as well.

Do you have any questions before we begin?

I will be starting the recorder now. Please let me know if at any point you would like me to turn off the recorder during the interview.

*Introductory questions*

1. To begin, could you introduce yourself and describe your role at the organization?
   1. What gender do you identify as and what are your preferred gender pronouns?
   2. What organization are you from?
   3. About how many people work at your organization? Where do most staff work from?
   4. What are your day-to-day activities at the organization?
2. Could you describe your experience with ASRH projects at the organization?
   1. How long have you been with the organization?
   2. What ASRH projects are you involved in?
   3. Where are they located?
3. What is your role on the ASRH project(s)?

*General questions*

1. Can you tell me more about your organization’s focus on ASRH?
2. How long has the organization been working in this area?
3. Generally, who funds the ASRH projects?
4. Can you tell me more about the project funded by Global Affairs Canada, ______ (name of specific project in discussion)?
   1. How is the funding distributed in the project?
   2. What was the process of building the ASRH project funded by GAC?
      1. How was the project developed?
         1. Who led the development? Who else was involved?
         2. Were adolescents involved? If so, could you tell me a bit about your experience collaborating with them? How were they involved?
         3. How were the priorities of the project set? Who set them?
         4. Did the call for proposals influence the project? If so, how?
      2. How is the project being implemented and evaluated?
         1. Who led/is leading the implementation and evaluation phase? Who else was involved (ex., Ministry of Health, Ministry of Gender, religious organizations, other NGOs, schools)?
         2. Were adolescents involved? If so, how?
         3. How is the project being evaluated? What are the measurements of impact?

*Stakeholder analysis*

1. Are you familiar with Canada’s Feminist International Assistance Policy (FIAP)?
   1. If so, how did you hear of it?
   2. What do you understand as the goals of the FIAP?

*If participants are not familiar with the FIAP, I will share the following and ask,* the FIAP is a policy that frames Canada’s funding for ASRH projects and emphasizes a feminist approach by targeting gender equality and empowerment of women and girls. Do you recognize any aspects of the FIAP in your work?

1. Can you tell me a bit about how the FIAP is relevant to your work?
   1. If the FIAP is not relevant, why not?
2. Is there an existing, similar policy to the FIAP within the implementation country of this project? How does this policy align or not with the FIAP?
   1. How has FIAP funding provided opportunities to do things that are aligned with country priorities (i.e., focus on gender, feminist approaches, poverty reduction)?
   2. Are there any differences between FIAP priorities and in-country priorities (i.e., around gender goals, feminist approaches, poverty reduction)?
3. In your opinion, what has shaped this ASRH project?
   1. Do organizational values/community goals/funding expectations/international or national government policies shape ASRH projects? If so, how?
   2. Has the FIAP, or aims of the FIAP, influenced or shaped this project? If so, how or in what ways?
      1. Has the FIAP influenced this project at certain stages, for example, at the development, implementation, or evaluation stage? If so, how?
4. What do you think are the main strengths and limitations of this ASRH project?
5. What do you think is the most important for funding organizations and policy makers to keep in mind with regards to supporting ASRH projects?
   1. How can funding organizations and policy makers better support ASRH projects?

*Final comments*

1. Is there anyone else from your organization that you would recommend I interview for this study?
2. Are there any GAC reports or similar project documents you would be open to sharing with me for this research?
3. Would you be interested in providing feedback on the data analysis codebook once it is provisionally developed from the interviews?
4. Is there anything else you would like to say about what we’ve talked about today?

*Debriefing*

Thank you very much for your time. If you’d like to get in touch with me after this interview, please do not hesitate to send an email. Would it be okay for us to contact you for clarification in the future? I have learned a lot today, thank you so much for sharing about your work and insights.
