# Supplementary material for: Contradictions and possibilities for change: Exploring stakeholder perspectives of Canada’s Feminist International Assistance Policy (FIAP) and their connection to a future for global health
Source: PLOS Glob Public Health. 2024 Nov 8;4(11):e0003877. doi: 10.1371/journal.pgph.0003877 (PMC11548757; doi:10.1371/journal.pgph.0003877)
Supplement: S2 Text — (DOCX) [file pgph.0003877.s002.docx]

## S2_Text

## Relevant Government Documents

**Methods to gather documents:** Document analysis is a method of qualitative research that consists of analyzing various documents including scholarly articles, policies, and institutional reports [1–3]. Document analysis of government documents was employed to verify the codebook of themes that was inductively developed from key informant interviews. Documents were defined as publicly available written material, which included information notes on SRH evaluation, government calls for proposals on ASRH, and government/NGO reports on ASRH and funding.

Documents were identified through manual electronic searches and with support from government-affiliated organizations and interview participants. An electronic search of documents was completed via Canadian government webpages and affiliate NGO websites (e.g., CanWaCH, Equality Fund). Documents were included if they were, 1) publicly available and, 2) discussed relevant GAC Call(s) for Proposals with a focus on ASRH (i.e., the Health and Rights Pall for proposals), government guidance on developing and/or evaluating ASRH projects, and/or GAC SRH key performance indicators or evaluation specific to SRH. Documents were gathered on an ongoing basis from May 2022-May 2023 by the lead student investigator and used in conjunction with the interview data.

**Table of relevant government documents:**

| **Document** | **Area(s) of relevance** |
| --- | --- |
| [Health and Rights Call for Proposals](https://www.international.gc.ca/world-monde/funding-financement/health_rights_women-sante_droits_femmes.aspx?lang=eng) | Development, funding, partnerships |
| [Health and Rights Call – Questions and Answers](https://www.international.gc.ca/world-monde/funding-financement/health_women-sante_femmes-QandA-QetR.aspx?lang=eng) | Development, implementation, evaluation, funding, partnerships |
| [Gender-based Analysis Plus](https://women-gender-equality.canada.ca/en/gender-based-analysis-plus/apply-to-work.html) | Development, evaluation (reporting) |
| [Gender Equality and Empowerment Measurement Tool](https://www.international.gc.ca/world-monde/assets/pdfs/funding-financement/gem-tool-introduction.pdf) | Development, evaluation (reporting) |
| [FIAP Indicators](https://www.international.gc.ca/world-monde/issues_development-enjeux_developpement/priorities-priorites/fiap_indicators-indicateurs_paif.aspx?lang=eng) | Development, funding, partnerships, evaluation (reporting) |
| [FIAP Toolkit](https://www.international.gc.ca/world-monde/funding-financement/gender_equality_toolkit-trousse_outils_egalite_genres.aspx?lang=eng) | Development, funding, partnerships, evaluation (reporting) |
| [Canada’s Policy for Civil Society Partnerships for International Assistance – A Feminist Approach](https://www.international.gc.ca/world-monde/issues_development-enjeux_developpement/priorities-priorites/civil_policy-politique_civile.aspx?lang=eng&_ga=2.148107511.1991931488.1686069023-1293255343.1686069022) | Development, funding, partnerships |
| [Development innovation overview](https://www.international.gc.ca/world-monde/issues_development-enjeux_developpement/priorities-priorites/development_innovation-innovation_developpement.aspx?lang=eng) | Development, funding, partnerships |
| Accountability Framework for Canada’s 10-Year Commitment to Global Health and Rights (available upon request) | Evaluation (reporting) |
| [GAC Results-Based Management Framework](https://www.international.gc.ca/world-monde/assets/pdfs/funding-financement/how-to-guide.pdf) | Evaluation (reporting) |
| [Technical Guidance Note on Developing Monitoring and Evaluation Plans for Global Health & Rights Programs](https://canwach.ca/learning/introducing-our-technical-guidance-note-on-developing-monitoring-and-evaluation-plans-for-global-health-rights-programs/) | Evaluation (reporting) |
| [SRHR Key Performance Indicators](https://canwach.ca/sites/default/files/2018-12/SGDE-EDRMS-%238260464-v2-SRHR_KPIs_FINAL.pdf) | Evaluation (reporting) |
| [SRHR KPI Methodological Notes](https://canwach.ca/sites/default/files/2019-02/v3-SRHR_KPI_Methodological_notes_partners_version%28ENGLISH%29.pdf) | Evaluation (reporting) |

##

References

1. Morgan H. Conducting a Qualitative Document Analysis. Qual Rep. 2022;27(1):64–77. doi:10.46743/2160-3715/2022.5044

2. Bowen, Glenn A. Document Analysis as a Qualitative Research Method. Qual Res J. 2009;9(2):27–40. doi:10.3316/QRJ0902027

3. Dalglish SL, Khalid H, McMahon SA. Document analysis in health policy research: The READ approach. Health Policy Plan. 2020;35(10):1424–31. doi:10.1093/heapol/czaa064
