# Supplementary material for: Contradictions and possibilities for change: Exploring stakeholder perspectives of Canada’s Feminist International Assistance Policy (FIAP) and their connection to a future for global health
Source: PLOS Glob Public Health. 2024 Nov 8;4(11):e0003877. doi: 10.1371/journal.pgph.0003877 (PMC11548757; doi:10.1371/journal.pgph.0003877)
Supplement: S1 Table — (DOCX) [file pgph.0003877.s003.docx]

**S1_Table**

**Thematic Analysis Codebook**

| **Focus area** | **Codes** | **Description** |
| --- | --- | --- |
| **Overview of organizations (role, SRHR focus)** | Oversight and support | Canadian/high-income-country (HIC) org does oversight and support of the project from Canada |
|  | Partnership/consortium model | Many partners, consortium model |
|  | Approach to SRHR | How do organizations approach SRHR |
|  | Recognized learning | Operations expertise rather than technical expertise, org acknowledges the opportunity for different experts in learning |
|  | Timeframe of SRHR focus | Focus on SRHR has emerged in the last 10 years; SRHR focus is connected to GAC funding |
| **Role of individuals** | Oversight, management and communication with donors | Role of the individuals, managerial/operations |
|  | Support | Role is to support, rather than prescribe ideas (i.e., non-hierarchical, accompanying) |
| **Overview of SRHR projects** | 3-4 pillar model | Usually a 3-4 pillar model of the project focused on advocacy, systems strengthening, info on SRHR etc. |
|  | GAC funded | Majority GAC funded, some in kind and/or from donors |
|  | Distribution of funding | Majority of money goes to partner organizations and implementation |
| **Project development (how, who, priorities)** | Call for proposals | Call for proposals influence in project |
|  | GAC guidelines | Strict GAC guidelines for funding; fit into project indicators to get funding |
|  | Timeframe for approval | Long timeframe of approval for projects |
|  | Consultation | Varying degrees of partner consultations in development process |
| **Project implementation (how, who)** | Partnerships | Project implementation is through partner orgs, WROs, YLOs |
|  | Collaborative | No line of direction from HIC org, HIC does GAC compliance and oversight/support/coordination |
|  | Challenges | Challenges with involving adolescents and community - difficult to do given cautiousness/risk of engaging minors, timeframes etc. |
|  | Decision making | Decision making in projects (i.e., who is involved, how, hierarchy?) |
| **Project evaluation (how, who)** | Compliance with GAC | MEL is all based on KPIs, GAC guidelines; strict guidelines; compliance with GAC is important for funding/future funding |
|  | MEL opportunities | Mostly baseline-midline-endline MEL plans; opportunity to expand MEL processes |
| **Perspectives of FIAP and its relevance** | A positive, very important step forward | FIAP is relevant and important to work/project |
|  | Frames all aspects of work | FIAP frames project aspects - logic model, project developed based on call, focus on neglected areas of SRHR because of FIAP |
|  | Contradictions | Misalignment of what FIAP calls for and what it supports; GAC not ready to action what FIAP demands; prescriptive to tell people what to do even if it's "good"; FIAP becomes almost performative |
| **Existing policies in countries of focus** | Tensions/differing goals | Some alignment with national policies/strategies; tension in some implementing countries (i.e., inclusion of LBGT, abortion etc.); opportunities to adapt based on in-country context |
|  | Safety risks | Have to consider risks of implementing FIAP values in countries where goals of FIAP are not welcomed |
|  | Bias towards donor | In country strategies are donor driven and policies like FIAP aren't needed for change, change will happen naturally and context specifically |
| **What has shaped ASRH project** | Org values and experience | Experience of org and individuals with expertise at org shapes project, existing org values and policies |
|  | FIAP | FIAP and its expectations shape projects (i.e., focus on 4 neglected areas of SRHR, gender transformation etc.) |
|  | Community | Voices of people implementing, government policies/strategies |
| **Strengths** | Project model and partnerships | Team, partners, consortium; the knowledge and expertise of partner organizations and team in country named as a strength |
|  | Learning across contexts | The opportunity to learn across contexts as projects are located in multiple countries |
|  | Multisectoral approach | Projects stretch across multiple sectors and organizations |
| **Limitations** | Timelines | Not enough time to implement or build partnerships with integrity, timelines are strict |
|  | Complex partnerships | Complexity of work with so many different partners |
|  | Strict funding | Funding requirements are strict and attached to KPIs |
|  | Relatively top down design | Design (based on timelines, funding etc.) is still relatively top down |
| **Future directions** | Remain flexible | Change takes time, capacity building takes time; funding and timelines should be more flexible and have ability to adjust to changing community priorities |
|  | Be bold | Innovation comes with risk, opportunity to embrace risk and possible failure; invest in progressive and innovative SRHR |
|  | Accountability | GAC to stick to commitments, accountability for safety of people implementing aspects of projects that could be dangerous in some contexts |
|  | Power and control | Don't be afraid to cede control and power; think about role, take a more reflexive approach, let go of power/control; engage in conversation with community |
