# Supplementary material for: Contradictions and possibilities for change: Exploring stakeholder perspectives of Canada’s Feminist International Assistance Policy (FIAP) and their connection to a future for global health
Source: PLOS Glob Public Health. 2024 Nov 8;4(11):e0003877. doi: 10.1371/journal.pgph.0003877 (PMC11548757; doi:10.1371/journal.pgph.0003877)
Supplement: S1 Fig — (DOCX) [file pgph.0003877.s004.docx]

## S1_Fig

## Overview of the Funding Process
