# Supplementary material for: Contradictions and possibilities for change: Exploring stakeholder perspectives of Canada’s Feminist International Assistance Policy (FIAP) and their connection to a future for global health
Source: PLOS Glob Public Health. 2024 Nov 8;4(11):e0003877. doi: 10.1371/journal.pgph.0003877 (PMC11548757; doi:10.1371/journal.pgph.0003877)
Supplement: Attachment — Submitted filename: Peer Review for PLOS Global Public Health Manuscript.pdf [file pgph.0003877.s005.pdf]

Peer Review for PLOS Global Public Health Manuscript: Contradictions and possibilities for change: Exploring stakeholder perspectives of Canada's Feminist International Assistance Policy (FIAP) and their connection to a future for global health

Title and Abstract: The title accurately reflects the content and focus of the study. The abstract is well-written, providing a concise summary of the study's objectives, methodology, key findings, and implications. It effectively captures the reader's interest and gives a clear overview of what to expect in the main text.

Introduction: The introduction provides a comprehensive background on the importance of adolescent sexual and reproductive health (ASRH) and the role of Canada's FIAP. The rationale for the study is clearly articulated, highlighting the gaps in existing research and the need for exploring stakeholder perspectives on FIAP's influence on ASRH projects. The research questions and objectives are clearly stated and relevant to the topic.

Methodology: The study employs a qualitative approach, using stakeholder analysis and qualitative description through eight interviews with key informants. This methodology is appropriate given the exploratory nature of the research questions. The selection of participants, data collection methods, and analysis procedures are adequately described. However, more details on the interview guide and how thematic analysis was conducted could enhance the reproducibility of the study. Also, the fact that they had to stay within Canada residents makes sense due to the COVID-19 pandemic, but it's a gap to miss the voices of the implementation partners.

Results: The results section is well-organized and presents the findings clearly and logically. The themes identified from the interviews are relevant and provide valuable insights into the influence of FIAP on ASRH projects. Using direct quotes from stakeholders enriches the narrative and supports the identified themes. The findings highlight the positive aspects of FIAP and the challenges faced in its implementation due to GAC regulations, providing a balanced view.

Discussion: The discussion section effectively interprets the findings concerning the study's objectives and existing literature. The authors provide a thoughtful analysis of the implications of their findings for policy and practice, particularly the need for more flexible funding processes, expanded monitoring and evaluation approaches, and increased timelines for consultation and partnership building. The connection to the conceptual framework for an imagined future for global health is well-articulated, adding depth to the discussion.

Conclusion: The conclusion briefly summarizes the key findings and their implications. The authors reiterate the potential of FIAP to promote equity and justice in ASRH projects, while also calling for ongoing re-imagination of funding structures and processes. The conclusion aligns with the study's objectives and provides a clear take-home message.

Strengths:

1. The study addresses a significant gap in the literature by exploring the practical impacts of FIAP on ASRH projects from the perspective of stakeholders.
2. The qualitative approach and use of direct quotes provide rich, contextualized insights.
3. The discussion connects the findings to broader theoretical frameworks, enhancing the study's contribution to the field.

Weaknesses:

1. The methodology section could benefit from more detailed information on the interview guide and thematic analysis process.
2. The study is based on a relatively small sample size (eight interviews), which may limit the generalizability of the findings.
3. On page 24 there's a mention of reparations vis a vis aid and there's not much analysis around that, which is a relevant example of the power dynamic between FIAP and the implementing partners, and how it's not addressing those, further than the GAC regulations.

Recommendations for Improvement:

1. Include more detailed descriptions of the interview guide and thematic analysis process in the methodology section or as an annex.
2. Discuss the limitations of the small sample size in more detail and consider ways to address this in future research.
3. Would love it if authors could dig deeper on the reparations vis a vis aid comment.

Overall Assessment: This study provides valuable insights into the influence of Canada's FIAP on ASRH projects and contributes to the broader discourse on global health policy and practice. The findings are relevant and timely, and the manuscript is well-written and structured. With minor revisions to enhance methodological transparency, this article can make a significant contribution to the field, hopefully influencing improvements on how GAC operates and how FIAP becomes more accountable to local stakeholders.

Oriana López Uribe
