# Supplementary material for: Contradictions and possibilities for change: Exploring stakeholder perspectives of Canada’s Feminist International Assistance Policy (FIAP) and their connection to a future for global health
Source: PLOS Glob Public Health. 2024 Nov 8;4(11):e0003877. doi: 10.1371/journal.pgph.0003877 (PMC11548757; doi:10.1371/journal.pgph.0003877)
Supplement: Attachment — Submitted filename: Response to reviewers.pdf [file pgph.0003877.s006.pdf]

Dear Editor and Reviewers,

Many thanks for the feedback. We are grateful for the insights and have made revisions accordingly. Please find our responses below. Relevant revisions are outlined in the manuscript on the indicated pages.

Reviewer	Comment	Authors' Response
**Editor**	Regarding Reviewer 2's overarching point, I believe the two components housed within the paper are a strength rather than a weakness. The length is acceptable. But given the concern described by Reviewer 2 about the 'two areas' of the paper, I would recommend seeking out ways to further integrate these areas as able throughout the manuscript.	We appreciate Reviewer 2's feedback on the two areas of the paper and have sought out ways to further integrate these areas within the manuscript. The integration of both areas is included throughout the paper as both tracked changes and existing language – in the introduction on lines 103-107, 119-121, 124-126, 129-130, 170-174; in the methods section on lines 287-289; in the results section on lines 301-303; in the discussion section on lines 589-596, 601-607, 620-623, 634-639, 657-659, 672-675, 701-702, 707-708, 711-714, 729-730 (and the related Figure 2), 733-736; and in the conclusion on lines 772-777.
**Reviewer 1**	This study provides valuable insights into the influence of Canada's FIAP on ASRH projects and contributes to the broader discourse on global health policy and practice. The findings are relevant and timely, and the manuscript is well-written and structured. With minor revisions to enhance methodological transparency, this article can make a significant contribution to the field, hopefully influencing improvements on how GAC operates and how FIAP becomes more accountable to local stakeholders.	We thank Reviewer 1 for their detailed and thoughtful feedback. Revisions have been made to the manuscript and are outlined below.
	Include more detailed descriptions of the interview guide and thematic analysis process in the methodology section or as an annex.	The interview guide has been included as Supplementary Information (S3 Appendix) indicated on p. 11 of the manuscript (line 235). We have also expanded on the themes of the analysis (p. 13, lines 277-289) and included the codebook that was used to complete the thematic analysis as Supplementary Information (S4 Appendix).
	Discuss the limitations of the small sample size in more detail and consider ways to address this in future research.	Thanks for bringing this point forward. We have added detail to the Limitations section on pp. 32-33 (lines 755-58 and 766-69) about the limitations of the small sample size and opportunities to address this in future research.

	Would love it if authors could dig deeper on the reparations vis a vis aid comment.	We appreciate the interest in this comment from a participant and share intrigue. This study was conducted through a Qualitative Description approach and as such, the quotes reflect directly what the participants said. This participant did not expand on what they meant by "reparations vis a vis aid" so we were unable to speculate further or expand about what this could mean. However, we have more directly referred to these ideas on pp. 30-31 while discussing reflexivity and the opportunity for funders to consider their funding within the context of power, history, and colonialism (lines 710-711 and 717-721).<br><br>Further research is encouraged to focus on the possibility of different funding structures and what this looks like given the colonial history of development aid.
**Reviewer 2**	In essence I found the paper way too long and really is directed at two areas---how FIAP is operationalized and influences ASRH projects within NGOs in eastern Africa and a precis of Ms Chadwicks dissertation on reimaging global health in a more equitable fashion. My suggestion is to consider splitting this paper into two or concentrate on describing FIAP and some of the difficulties in operationalizing it. I would also expand the number of interviews done. As I read it there were only 8 interviews completed due to COVID issues and these were often done not in the field because of COVID limitations. I found the concept of FIAP Canadian approach fascinating and was unaware of its existence as I am sure many readers outside of Canada would be. For readers not that familiar with this mandate and its impact on NGOs, that in itself makes for an interesting paper	Thank you for your valuable feedback. We have addressed the specific suggestions below.
	Line 22—What percentage of key informants refused to participate?. Was there possible selection bias that occurred? What percentage of key informants were queried thru on the ground participants or thru the Canadian NGO office.	We outline the process for recruitment and resulting participation of individuals on p. 10-11 (lines 206-223). Participants were recruited virtually through CanWaCH which has direct contact with Canadian and implementing NGOs. Given that 8/11 individuals participated in the

		interviews, 27% declined to participate whereas 73% participated.<br><br>To mitigate selection bias, the inclusion criteria was discussed thoroughly with the team and in partnership with colleagues at CanWaCH. Given that the FIAP was only introduced in 2017, there is a limited number of projects in sub-Saharan Africa and even more specifically, that focus on ASRH and are led by NGOs. The study includes these projects.
	Lines 320-326 Paragraph designates 20 million dollars as the cutoff for NGO participation yet in your table there are 4 projects over 20 million.	Thank you for bringing this forward. We have clarified the language on p. 15, line 324 as $20million was the general indicator to demonstrate funding rather than the cut off.
	Lines 353 -356 Important to emphasize the need for outcome parameters to be developed together and not only mandated by Canada funding requirements. Define "outcome harvesting" better.	Outcome harvesting has been further defined on p. 17, lines 360-364.
	Line 480-482 grammar of quote confusing---"Neither we can accept......	This is a direct quote from a participant and, as per the Qualitative Description approach which aims to honour participants' perspectives as valuable end-products in themselves, we have aligned the quotes in text to report what participants said verbatim. Although perhaps slightly grammatically confusing, we believe the meaning of the quote is still captured.
	Line 597 What is the restrictive nature of GAC funding. (I know in supplement) but you might highlight to the reader some of the worst requirements and auditing which the grassroot organizations are having difficulties with.	We have included a brief note on some of the restrictive aspects of GAC funding on p. 26 (lines 607-609).
	Line 701-704. What are photovoice and body mapping—define	Definitions of body mapping and photovoice have been included on p. 31 (lines 717-721).
	Line 736-Limitations of COVID and using Canadian NGO partners may have limited the ability of investigators to build trust and get honest answers. Would be helpful to end with looming political issues such as addressing homosexuality, gender violence and other charged subjects	Thanks for bringing this point forward. We have added detail to the Limitations section on pp. 32-33 (lines 744-45 and 767-69) about the complexity of SRH as a topic.
